# Trial Enrollment and Survival Disparities Among Patients With Advanced Epithelial Ovarian Carcinoma

**DOI:** 10.1001/jamanetworkopen.2025.38648

**Published:** 2025-10-22

**Authors:** Caitlin Ruth Johnson, Alex A. Francoeur, Amandeep Grewal, Natalie L. Ayoub, Michael T. Richardson, Daniel S. Kapp, Kathleen M. Darcy, Chunqiao Tian, John K. Chan

**Affiliations:** 1 Sutter Health, Walnut Creek, California; 2 Department of Obstetrics and Gynecology, University of California, Irvine, Orange; 3 Department of Obstetrics and Gynecology, University of California, Los Angeles; 4 Department of Radiation Oncology, Stanford University School of Medicine, Palo Alto, California; 5 Gynecologic Cancer Center of Excellence, Gynecologic Surgery and Obstetrics, Uniformed Services University of the Health Sciences, Walter Reed National Military Medical Center, Bethesda, Maryland; 6 The Henry M. Jackson Foundation for the Advancement of Military Medicine Inc., Bethesda, Maryland

## Abstract

**Importance:**

Racial differences in epithelial ovarian cancer (EOC) might result in survival inequities.

**Objective:**

To evaluate enrollment and outcomes by race in Gynecologic Oncology Group (GOG) randomized clinical trials (RCTs) among patients with EOC.

**Design, Setting, and Participants:**

This cohort study used ancillary data from completed RCTs using protocols GOG-111, GOG-114, GOG-158, and GOG-172 under a data sharing agreement with National Research Group Oncology. Patients with stage III or IV EOC in first-line RCT protocol GOG-111 had suboptimally resected disease, whereas those in GOG-114, GOG-158, or GOG-172 had optimally resected disease. RCTs were conducted and published between 1996 and 2006, and data for this study were analyzed in August 2024.

**Exposure:**

Race was categorized as Asian, Black or African American (Black), or White or Caucasian (White). Patients of other races were excluded. Spanish ethnicity and additional details regarding residual disease status were not available for analysis.

**Main Outcomes and Measures:**

Overall survival (OS) was the primary end point and progression-free survival (PFS) was the secondary end point, evaluated using multivariable Cox proportional-hazards modeling and log-rank testing. Statistical significance was set at *P* < .05.

**Results:**

This study included 1903 evaluable participants, of whom 35 (1.84%) self-identified as Asian, 121 (6.36%) as Black, and 1747 (91.80%) as White. Black patients had lower OS (median [IQR], 36.8 [19.2-73.4] months) than Asian (50.9 [23.9-109.2] months) or White (48.4 [24.5-93.4] months) patients (*P* = .03), with a higher risk of death than White patients (adjusted hazard ratio, 1.30; 95% CI, 1.06-1.59; *P* = .01). PFS and adjusted risk of disease progression were statistically similar across racial groups. Median (IQR) PFS was 18.9 (9.7-84.6), 18.0 (9.1-34.0), and 19.7 (11.5-43.3) months among Asian, Black, and White patients, respectively (*P* = .08). Adjusted risk of disease progression was similar for Black patients compared with White patients (adjusted hazard ratio, 1.21; 95% CI, 1.00-1.47; *P* = .06).

**Conclusions and Relevance:**

In this cohort study, Black and Asian patients were underrepresented in RCT trial populations. Black patients had lower OS than White and Asian patients but similar PFS. Equitable enrollment in clinical trials ensures access to cutting-edge treatments and can lead to outcomes comparable to those of White counterparts. Sustained efforts to improve RCT diversity remain essential to long-term equity in cancer care and survival.

## Key Points

**Question:**

Do enrollment and survival outcomes vary by race among patients participating in randomized clinical trials?

**Finding:**

In this cohort study analyzing data from 4 completed randomized phase 3 trials with 1903 evaluable participants, Black patients made up 6.4% and Asian patients represented 1.8% of these trials’ populations. Overall survival (the primary end point) was lower among Black patients compared with White and Asian patients, whereas progression-free survival rates were not significantly different among racial groups.

**Meaning:**

A lack of diversity was observed in clinical trials, highlighting the need for more equitable enrollment and outcomes for all patients.

## Teaser

This cohort study examines racial differences in randomized clinical trial enrollment and overall survival among patients with advanced epithelial ovarian carcinoma.

## Introduction

Prospective randomized clinical trials are integral in determining the standard of care when treating patients with any oncologic malignancy. A substantial body of literature confirms that racial and ethnic disparities persist in clinical trial recruitment and participation.^1,2,3,4^ Ovarian cancers contribute to a high proportion of patient morbidity and mortality among those with gynecologic cancers in the US. Patients with ovarian cancer contribute to most (64%) clinical trials conducted investigating treatments of gynecologic cancer.^5,6^ Racial disparities in clinical trials for ovarian cancer are more pronounced compared with other gynecologic malignancies, such as cervix and uterine cancer.^7,8,9^

Although many studies have reported on the lack of diverse representation in clinical trials on gynecologic malignant neoplasms, fewer have examined the actual clinical outcomes of patients enrolled on these trials by race and ethnicity.^10,11^ This study aimed to evaluate outcomes from prospective randomized clinical trials (RCTs) from the Gynecologic Oncology Group (GOG) under a data sharing agreement with National Research Group (NRG) Oncology, focusing on differences by race while controlling for non-Hispanic ethnicity. We hypothesized that in a controlled setting in which all patients met standardized eligibility criteria, received a controlled treatment, and were followed consistently for prespecified end points, we would not detect racial differences in either progression-free survival (PFS) or overall survival (OS) outcomes.

## Methods

This cohort study was a retrospective ancillary analysis of RCT data collected from 4 pivotal GOG trials involving patients undergoing first-line treatment for advanced epithelial ovarian cancer (EOC). All patients had histologically confirmed stage III or IV EOC. Per the original trial inclusion criteria, patients on RCT protocol GOG-111 had suboptimally resected disease, defined as having a residual tumor size of 1 cm or greater, and patients on GOG-114, GOG-158, or GOG-172 had optimally resected disease, defined as having either complete gross resection or a residual tumor size of less than 1 cm. These 4 RCTs were conducted and published between 1996 and 2006.^12,13,14,15^ The GOG Foundation, a nonprofit organization funded by both the National Cancer Institute and industry partners, conducted these trials to advance translational and clinical research in the field of gynecologic cancer.^16^ Patients enrolled in these RCTs underwent surgical staging followed by adjuvant chemotherapy. Patients were required to have a GOG performance status of 0 to 2, and patients with prior radiation or neoadjuvant chemotherapy were excluded from participation. Performance status before the start of chemotherapy was graded according to GOG criteria: 0 (full ambulatory with no restrictions), 1 (restricted in physically strenuous activity but ambulatory and able to perform light activity), and 2 (ambulatory and capable of self-care but unable to perform work activity). More detailed information regarding inclusion and exclusion criteria is available in the trial protocol publications for these RCTs.^12,13,14,15^ The data were acquired from the NRG Oncology through an approved data sharing application and agreement executed in September 2021. The present study was reviewed and approved by Sutter Health. This study was an ancillary analysis of previously collected data; therefore, informed consent from patients was not required. This study followed the Strengthening the Reporting of Observational Studies in Epidemiology (STROBE) reporting guidelines for cohort studies.

Self-reported race was categorized as Asian, Black or African American (Black), or White or Caucasian (White). Patients who self-identified as another race were excluded from the analysis due to inconsistent or limited inclusion in these trials. Age was categorized at the median (57 years) into 2 groups: 18 to 56 and 57 to 86 years. Stage III disease was defined as disease confined to the abdomen, and stage IV disease was defined as having either spread outside the abdomen or into the parenchyma of abdominal organs, such as the liver or spleen. Histological subtypes of epithelial cancer and tumor grade were confirmed centrally by the study’s pathology committee.

### Statistical Analysis

Categorical comparisons among different racial groups were tested using a χ^2^ test or Fisher exact test. Fisher exact test was used when a cell count was less than 5. The clinical covariates were not evaluated as continuous variables. The primary end point for this cohort study was OS, defined as the time in months from clinical trial enrollment to all-cause death for events or to last contact for censored patients who were alive at last contact. The secondary end point was PFS, defined as the time in months from clinical trial enrollment to first disease recurrence, progression while alive, or death, or to last contact for censored patients who were alive and free from recurrence or disease progression. PFS and OS distributions were estimated using the Kaplan-Meier method and compared using a log-rank test. Adjusted hazard ratios (aHRs) and 95% CIs were calculated to estimate risk of all-cause death or disease progression using multivariable Cox regression modeling, with adjustments for race, age group, performance status, disease stage, tumor histology, tumor grade, the aggregate between debulking status and RCT enrollment, and chemotherapy treatment route. The Schoenfeld residual test was used to confirm the proportional hazards assumption in Cox regression models. Statistical significance was set at a 2-sided *P* < .05. We did not include corrections for multiple testing in these analyses. All statistical analyses were performed in August 2024 using SAS, version 9.4 (SAS Institute), with follow-up extending up to 10 years from enrollment and randomization on the first-line treatment trial.

## Results

Of 1903 evaluable patients with advanced EOC enrolled in 4 prospective RCTs, 35 (1.84%) self-identified as Asian, 121 (6.39%) as Black, and 1747 (91.80%) as White. The median (range) age at diagnosis was 57 (18-86) years, with most having a GOG performance score of 0 or 1, stage III disease, and serous histology. Patient characteristics are summarized in Table 1 and Table 2. Patients in GOG-111 had suboptimally resected disease, whereas those in GOG-114, GOG-158, and GOG-172 had optimally resection disease. All patients across the 4 RCTs received platinum-based chemotherapy (Table 1). In this study, participants from both the experimental and standard-of-care control arms were included in the analysis. The distribution of racial groups was well balanced in both trial arms, allowing them to be compared across study arms. RCT details are displayed in Table 1, with additional information previously reported.^12,13,14,15^

**Table 1.** Summary of the Treatment Arms in the 4 First-Line Randomized Clinical Trials

Trial^a^	Arm		Patients, No.		
			Asian (n = 35)	Black (n = 121)	White (n = 1747)
	Experimental	Control			
GOG-111	Paclitaxel 135 mg/m^2^ IV and cisplatin 75 mg/m^2^ IV	Cyclophosphamide 740 mg/m^2^ IV and cisplatin 75 mg/m^2^ IV	4	32	343
GOG-114	Paclitaxel 120 mg/m^2^ as a 96-h infusion and cisplatin 75 mg/m^2^ on day 5	Paclitaxel 135 mg/m^2^ as a 24-h infusion and cisplatin 75 mg/m^2^ IV	4	29	412
GOG-158	Paclitaxel 175 mg/m^2^ IV and carboplatin AUC 7.5 IV	Paclitaxel 135 mg/m^2^ IV and cisplatin 75 mg/m^2^ IV	17	50	676
GOG-172	Paclitaxel 135 mg/m^2^ IV and cisplatin 100 mg/m^2^ IP and paclitaxel 60 mg/m^2^ IP on day 8	Paclitaxel 135 mg/m^2^ IV and cisplatin 75 mg/m^2^ IV	10	10	316

Abbreviations: AUC, area under the curve; GOG, Gynecologic Oncology Group; IV, intravenous chemotherapy; IP, intraperitoneal chemotherapy.

^a^ The citation for the primary randomized clinical trial reference was as follows: GOG-111,^13^ GOG-114,^12^ GOG-158,^14^ and GOG-172.^15^

**Table 2.** Characteristics of Patients With Advanced Epithelial Ovarian Cancer

Characteristic^a^	Patients, No. (row %)			*P* value
	Asian (n = 35)	Black (n = 121)	White (n = 1747)	
Age at diagnosis, y				
18-56	27 (2.95)	69 (7.55)	818 (89.50)	<.001
57-86	8 (0.81)	52 (5.25)	929 (93.93)	
GOG performance status				
Score 0	4 (2.12)	19 (10.05)	166 (87.83)	.01
Score 1	9 (1.14)	37 (4.69)	743 (94.17)	
Score 2	22 (2.38)	65 (7.03)	838 (90.59)	
Stage of disease				
Stage III	32 (1.81)	108 (6.09)	1632 (92.10)	.20
Stage IV	3 (2.29)	13 (9.92)	115 (87.79)	
Tumor grade				
Grade 1	1 (0.54)	17 (9.19)	167 (90.27)	.14
Grade 2	15 (2.05)	52 (7.12)	663 (90.82)	
Grade 3	19 (1.92)	52 (5.26)	917 (92.81)	
Tumor histologic subtype				
Serous carcinoma	25 (1.82)	88 (6.39)	1264 (91.79)	.10
Endometrioid carcinoma	4 (2.08)	14 (7.29)	174 (90.63)	
Clear cell carcinoma	1 (1.72)	1 (1.72)	56 (96.55)	
Mucinous carcinoma	1 (2.22)	8 (17.78)	36 (80.00)	
Mixed epithelial carcinoma	1 (0.75)	5 (3.73)	128 (95.52)	
Other carcinoma subtypes	3 (3.09)	5 (5.15)	89 (91.75)	
Randomized clinical trial^b^				
GOG-111 (suboptimal-resected)	4 (1.06)	32 (8.44)	343 (90.50)	.02
GOG-114 (optimal-resected)	4 (0.90)	29 (6.52)	412 (92.58)	
GOG-158 (optimal-resected)	17 (2.29)	50 (6.73)	676 (90.98)	
GOG-172 (optimal-resected)	10 (2.98)	10 (2.98)	316 (94.05)	
Chemotherapy treatment route				
Intravenous	30 (1.99)	101 (6.71)	1375 (91.30)	.29
Intraperitoneal	5 (1.26)	20 (5.04)	372 (93.70)	

Abbreviation: GOG, Gynecologic Oncology Group.

^a^ All clinical covariates were evaluated as categorical rather than continuous variables.

^b^ The citation for the primary randomized clinical trial reference was as follows: GOG-111,^13^ GOG-114,^12^ GOG-158,^14^ and GOG-172.^15^

**Figure 1.**
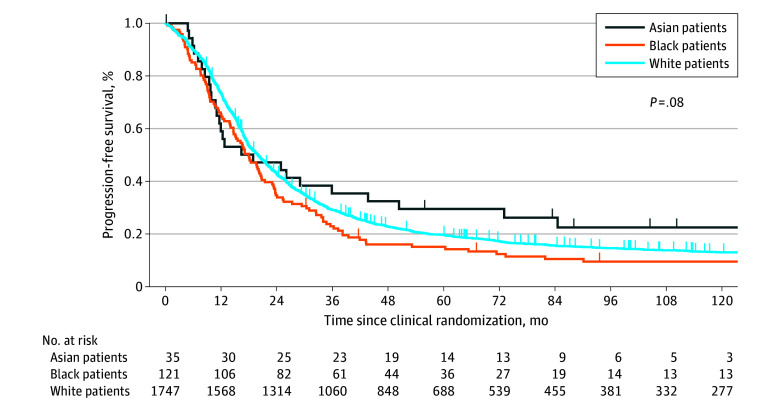
Progression-Free Survival Among Patients Treated in Gynecologic Oncology Group (GOG) Randomized Clinical Trials by Race Progression-free survival was evaluated using the Kaplan-Meier method and compared using a log-rank test among Asian, Black, and White patients. The x-axis represents time in months from enrollment in the randomized clinical trial protocols GOG-111, GOG-114, GOG-158, or GOG-172. The y-axis represents the proportion of patients who were progression-free at last contact. The study included 35 Asian patients (26 who experienced disease progression and 9 who were progression-free at last contact), 121 Black patients (111 who experienced disease progression and 10 who were progression-free at last contact), and 1747 White patients (1513 who experienced disease progression and 234 who were progression-free at last contact). The patients at risk are provided below the x-axis.

Asian patients were diagnosed at a younger age compared with other racial groups (18-56 years: 27 [2.95%] Asian, 69 [7.55%] Black, and 818 [89.50%] White; 57-86 years: 8 [0.81%] Asian, 52 [5.25%] Black, and 929 [93.93%] White; *P* < .001) (Table 2). Black and Asian patients were more likely to have a lower GOG performance score compared with White patients, with a performance score of 2 observed in 22 Asian (62.86%), 65 Black (53.72%), and 838 White (47.97%) patients, respectively (*P* = .01). However, no statistically significant association was found between race and disease stage, tumor histology, or tumor grade. There was no difference in the chemotherapy treatment route (intravenous vs intraperitoneal) by race (Table 2). Older patients had a higher adjusted risk of death compared with younger patients (aHR, 1.21; 95% CI, 1.09-1.34; *P* < .001). A higher GOG performance score was associated with an increased risk of death (aHR, 1.17; 95% CI, 1.05-1.31; *P* < .001 for score 1 vs 0 and aHR, 1.46; 95% CI, 1.22-1.74; *P* = .004 for score 2 vs 0). Mucinous (aHR, 3.88; 95% CI, 2.82-5.35; *P* < .001) and clear cell (aHR, 1.47; 95% CI, 1.09-1.99; *P* = .01) carcinomas were associated with worse survival compared with serous histology. Patients with optimally resected disease had better outcomes than those with suboptimal resection (Table 3). Additional factors are listed in Table 3. We then analyzed the secondary end point, PFS, and found a median (IQR) PFS of 18.9 (9.7-84.6) months among Asian patients, 18.0 (9.1-34.0) months among Black patients, and 19.7 (11.5-43.3) months among White patients (*P* = .08) (Figure 1). After adjusting for other prognostic factors (age, performance, disease stage, tumor histology, tumor grade, RCT protocol, and chemotherapy treatment route), the disease progression was similar between Black and White patients (aHR, 1.21; 95% CI, 1.00-1.47; *P* = .06) and Asian and White patients (aHR, 0.89; 95% CI, 0.60-1.32; *P* = .58).

**Table 3.** Hazard Ratios (HRs) for Risk of All-Cause Death and Disease Progression Among Patients With Advanced Epithelial Ovarian Cancer

Variable	Risk of death (primary end point)		Risk of disease progression (secondary end point)	
	Adjusted HR (95% CI)	*P* value	Adjusted HR (95% CI)	*P* value
Race (reference group, White)				
Black	1.30 (1.06-1.50)	.01	1.21 (1.00-1.47)	.06
Asian	0.99 (0.66-1.48)	.94	0.89 (0.60-1.32)	.58
Age at diagnosis, y (reference group, 18-56)				
57-86	1.21 (1.09-1.34)	<.001	1.10 (0.10-1.21)	.06
GOG performance score (reference group, 0)				
1	1.17 (1.05-1.31)	<.001	1.10 (0.99-1.22)	.08
2	1.46 (1.22-1.74)	.004	1.16 (0.978-1.38)	.09
Stage of disease (reference group, 3)				
4	0.97 (0.78-1.22)	.81	1.12 (0.90-1.39)	.33
Tumor histologic subtype (reference group, serous carcinoma)				
Mucinous carcinoma	3.88 (2.82-5.35)	<.001	2.79 (2.04-3.84)	<.001
Clear cell carcinoma	1.47 (1.09-1.99)	.01	1.08 (0.80-1.45)	.61
Mixed epithelial carcinoma	1.04 (0.85-1.27)	.71	0.92 (0.76-1.12)	.41
Endometrioid carcinoma	0.80 (0.67-0.96)	.02	0.73 (0.61-0.87)	<.001
Other subtypes	0.73 (0.57-0.93)	.01	0.69 (0.55-0.87)	.002
Tumor grade (reference group, 1)				
2	1.63 (1.33-2.00)	<.001	1.05 (0.95-1.17)	.36
3	1.49 (1.22-1.83)	<.001	0.71 (0.59-0.86)	<.001
Debulking and randomized clinical trial protocol (reference group, GOG-111 [suboptimal-resected])^a^				
GOG-114 (optimal-resected)	0.55 (0.45-0.66)	<.001	0.69 (0.57-0.83)	<.001
GOG-158 (optimal-resected)	0.58 (0.50-0.68)	<.001	0.73 (0.63-0.86)	<.001
GOG-172 (optimal-resected)	0.56 (0.46-0.68)	<.001	0.70 (0.58-0.85)	<.001
Chemotherapy treatment route (reference group, intraperitoneal)				
Intravenous	1.15 (0.98-1.35)	.10	1.18 (1.01-1.38)	.04

Abbreviation: GOG, Gynecologic Oncology Group.

^a^ The citation for the primary randomized clinical trial reference was as follows: GOG-111,^13^ GOG-114,^12^ GOG-158,^14^ and GOG-172.^15^

**Figure 2.**
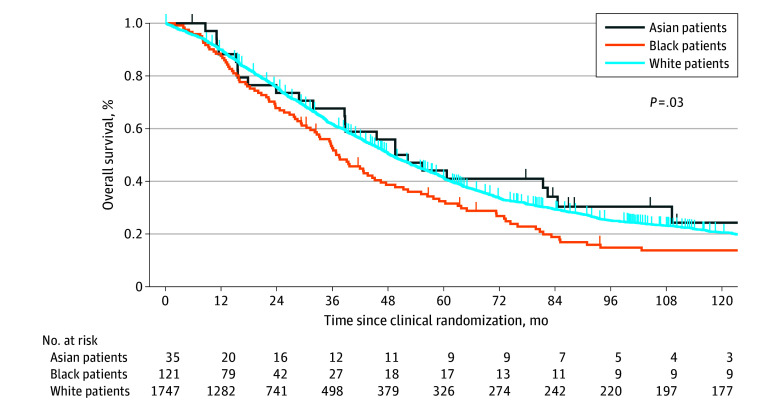
Overall Survival Among Patients Treated in Gynecologic Oncology Group (GOG) Randomized Clinical Trials by Race Overall survival was evaluated using the Kaplan-Meier method and compared using a log-rank test among Asian, Black, and White patients. The x-axis represents time in months from enrollment in the randomized clinical trial protocols GOG-111, GOG-114, GOG-158, or GOG-172. The y-axis represents the proportion of patients who are alive at last contact. The study included 35 Asian patients (24 who died and 11 who were still alive at last contact), 121 Black patients (102 who died and 19 who were still alive at last contact), and 1747 White patients (1380 who died and 367 who were alive at last contact). The patients at risk are provided below the x-axis.

Next, we evaluated OS (the primary end point). Black patients had significantly lower OS than White and Asian patients (Figure 2). Median (IQR) OS was 36.8 (19.2-73.4) months among Black patients compared with 50.9 (23.9-109.2) months and 48.4 (24.5-96.4) months among Asian and White patients, respectively (*P* = .03). Furthermore, the risk of death was higher among Black patients than White patients (aHR, 1.30; 95% CI, 1.06-1.59; *P* = .01) and similar among Asian and White patients (aHR, 0.99; 95% CI, 0.66-1.48; *P* = .94) after adjusting for the covariates in Table 3.

## Discussion

Ovarian cancer is one of the leading causes of death among women diagnosed with a gynecologic malignant neoplasm.^17^ Despite advancements in treatment, improvement has not been demonstrated equally among various racial groups.^18,19^ Previous literature has shown a lack of representation among racial groups in clinical trials involving gynecological malignant neoplasms.^6,7,8^ Studies show that oncologic patients enrolled in clinical trials have improved survival compared with those who are not.^20,21^ Conversely, other studies have reported that minorities have worse health outcomes despite enrolling in clinical trials.^3,22^ A study by Albain et al^22^ demonstrated that Black patients with sex-specific cancers, such as breast cancer or prostate cancer, had decreased survival compared with their White counterparts. Most of these studies did not address gynecologic cancers in particular, such as ovarian cancer.

We conducted a large ancillary data study of survival outcomes by race among patients with advanced EOC in collaboration with NRG Oncology. These patients participated in 4 first-line RCT protocols conducted and published by the GOG. In our study, no significant difference in progression-free survival was observed between Black and White patients, even after adjusting for important prognostic factors, such as age, disease stage, tumor histology, and chemotherapy treatment route. Asian patients had a similar adjusted risk of all-cause death compared with White patients in this aggregated cohort. Although disparities in cancer outcomes persist in the general population, some research suggests that these disparities can be reduced or eliminated when patients participate in trials. A study of patients with renal cell carcinoma did not find a difference in time to progression among different racial groups enrolled in clinical trials.^22^ In this study of 122 patients, Black patients survived 4.6 months shorter than their White counterparts (*P* < .01).^22^

The PFS findings of our study align with 2 previous studies that analyzed outcomes by race in similar GOG clinical trials.^10,11^ Farley et al^10^ compared patients treated with cisplatin and paclitaxel in RCT protocols GOG-111, GOG-114, GOG-132, GOG-152, GOG-158, GOG-162, and GOG-172, finding no differences in PFS or OS between Black and White patients. A study by Winter et al^11^ analyzed outcomes from RCT protocols GOG-111, GOG-114, GOG-132, GOG-152, GOG-158, and GOG-172, but limited the analysis to experimental arms in both GOG-152 and GOG-158.^11^ This study also demonstrated no difference in PFS and OS between Black and White patients. On the contrary, a study by Kim et al^23^ evaluated racial survival disparities in breast cancer clinical trials, showing that Black participants were more likely to have inferior disease-free survival outcomes compared with White participants, despite being enrolled in the same clinical trial.

We attribute the similar PFS across races to the fact that PFS more accurately reflects treatment effect in clinical trials. Trials are protocol-driven, involve favorable patient selection, include frequent assessments, and provide access to cutting-edge therapies.^24^ Additionally, clinical trials closely manage drug side effects, ensuring strict adherence to treatment plans, which minimizes deviation from trial design. This controlled environment helps eliminate disparities seen in the general population.

Although we showed that the PFS was comparable among these racial groups, our data subsequently demonstrated that OS was lower among Black patients compared with White patients. Our analysis includes participants from both the control and treatment arms for each racial group. The decline in OS among Black patients might be due to their transition from clinical trials to community-based treatment after disease progression. Without the rigorous protocols and oversight offered by clinical trials, treatment, follow-up, and surveillance in the community can vary. Analyses of clinical trial data demonstrate that Black patients still have lower OS despite being on the same clinical trial as their White counterparts.^3^ Furthermore, multiple database studies support this perspective, showing lower OS in Black patients with gynecologic malignant neoplasms that are treated in community settings.^25,26^ The authors attribute the differences in PFS and OS to rigorous effect management in trials vs variations in posttrial therapies. We suggest that Black patients might receive treatment that deviates from standards of care after recurrence, once outside a clinical trial.^18,19^

Participation in clinical trials has been associated with improved survival outcomes for patients with gynecologic malignancies.^27^ To assure equitable access and appropriate generalizability of results, it is important that the clinical trial represents the diversity of the population with the disease being studied. However, disparities in enrollment among minority populations in gynecologic cancer trials persist. A recent study demonstrated that Black women continue to be underrepresented in ovarian cancer trials, whereas Asian and Hispanic women are underrepresented in cervical, uterine, and ovarian cancer clinical trials.^28^ This trend is reflected in our study, in which Black patients represented only 6% of the cohort, compared with the 13.7% reported in the US Census Bureau.^29^ Barriers to recruiting diverse populations in clinical trials can be analyzed across various levels: systemic, individual, and interpersonal.^30^ Clinical trial shortages and academic hubs limit minority access, as underinsured patients often rely on community hospitals, and strict eligibility criteria disproportionately exclude Black or older patients.^30,31,32^ Clinician biases and limited trial awareness in community settings further reduce enrollment.^33,34,35^ These systemic and individual barriers perpetuate underrepresentation in cancer research.

Additionally, patient mistrust of the medical system has a historical basis from systematic exploitation of minority populations.^31^ One study by Mouton et al^36^ examined barriers to Black patients’ enrollment in cancer clinical trials. The authors reported that only 28% of Black women viewed clinical research in the US as ethical, and one-third of Black participants stated that it was difficult to trust scientists. Lastly, a review of more than 2000 gynecologic oncology clinical trials since 2007 revealed that only 40% reported information on race and ethnicity, calling attention to a critical gap in data transparency and accountability.^6^ In summary, further research and education are needed to build trust and improve representation in clinical trials. This inclusion will foster equitable access and outcomes for all patients. Increasing minority enrollment in clinical trials requires a multifaceted approach, including system-level interventions, infrastructure support, and coverage for uninsured patients.^30,35,36^ It is important to recognize that racial disparities often stem from health care access, systemic racism, and social determinants of health, rather than inherent biological differences.^31,37,38^

Future directions include expanding representation in clinical trials to include a diverse patient population that is representative of the study population and powering prospective trial designs for disparity analyses. Mandatory reporting of race and ethnicity is crucial in ensuring that these efforts are tracked systematically and that disparities can be adequately addressed. These types of changes will allow future trials to potentially be powered to evaluate differences in outcomes based on race and ethnicity. These data are important to assess root causes and make meaningful changes that will positively impact survival data for patients with gynecologic malignancies.

### Strengths and Limitations

This study has multiple strengths. This study analyzed data from multiple completed phase 3 RCTs conducted by the GOG group, which had a large sample size and high event rate. The trials span 11 years, providing a large time frame for analysis and long-term follow-up extending to 10 years. The randomized nature of the data allows for appropriate controlling of confounders.

Our study has limitations that might impact the interpretation of the findings and limit their generalizability. First, cause of death, which was documented for these 4 RCTs but was not provided by NRG Oncology as a clinical covariate for this ancillary data study, represents a limitation. Lacking this information prevented us from separating cause of death attribution into treatment-related, cancer-related, cancer- and treatment-related, other cause, or unknown cause. Ethnicity was not provided by NRG Oncology, which limits our ability to identify potential disparate outcomes between Hispanic and non-Hispanic populations. The presence of gross residual disease within the optimally resected trials (GOG-114, GOG-158, and GOG-172) and the presence of measurable disease for the suboptimally resected trial (GOG-111) were not provided by NRG Oncology, which precluded our ability to evaluate residual disease status in this ancillary data analysis. Although the trials included in our ancillary data analysis cohort were prospective in nature, the original trials were not powered to answer the specific questions assessed in this study. For example, this cohort study had an insufficient number of Asian patients to support a powered comparison with White patients. In addition, information about Asian ancestral subsets was not available; therefore, we could not examine outcomes by country of origin. These RCTs primarily enrolled White patients, limiting our ability to analyze differences in other racial and ethnic groups.

## Conclusions

In this cohort study of more than 1900 patients in RCTs for advanced EOC, Black and Asian patients were underrepresented compared with the general population estimates in the US, and Black patients had worse survival outcomes compared with White and Asian patients, despite having similar PFS. This study underscores the need to achieve equitable representation in clinical trials and outcomes for all patients with advanced EOC.

## Notes

Supplement.
Data Sharing Statement
