# Supplementary material for: Trial Enrollment and Survival Disparities Among Patients With Advanced Epithelial Ovarian Carcinoma
Source: JAMA Netw Open. 2025 Oct 22;8(10):e2538648. doi: 10.1001/jamanetworkopen.2025.38648 (PMC12547579; doi:10.1001/jamanetworkopen.2025.38648)
Supplement: Supplement. — Data Sharing Statement [file jamanetwopen-e2538648-s001.pdf]

Data Sharing Statement

Johnson. Trial Enrollment and Survival Disparities Among Patients With Advanced Epithelial Ovarian Carcinoma. *JAMA Netw Open*. Published October 22, 2025.
doi:10.1001/jamanetworkopen.2025.38648

Data
Data available: No

Additional Information
Explanation for why data not available: HIPAA-compliant data files for this investigation were acquired through a restricted access approval process with NRG Oncology with a data sharing agreement that prohibits sharing.
